# Gout and the risk of dementia: a nationwide population-based cohort study

**DOI:** 10.1186/s13075-015-0642-1

**Published:** 2015-05-28

**Authors:** Jen-Yee Hong, Tzuo-Yun Lan, Gau-Jun Tang, Chao-Hsiun Tang, Tzeng-Ji Chen, Hsiao-Yi Lin

**Affiliations:** Division of Allergy-Immunology-Rheumatology, Veterans General Hospital-Taipei, No.201, Sec. 2, Shipai Rd, Beitou District, Taipei, 11217 Taiwan; Institute of Hospital and Health Care Administration, National Yang-Ming University, Taipei, Taiwan; School of Health Care Administration, College of Medicine, Taipei Medical University, Taipei, Taiwan; Division of Family Medicine, Veterans General Hospital-Taipei, Taipei, Taiwan; National Yang-Ming University School of Medicine, Taipei, Taiwan

## Abstract

**Introduction:**

Uric acid was proposed to have anti-oxidant property and possible neuroprotective effects. We examined the association between gout and dementia with population database.

**Methods:**

The study utilized the claims data from the nationwide representative sample of Taiwan National Health Insurance Research Database (NHIRD). We ascertained patients with gout and dementia covering vascular and non-vascular (including Alzheimer’s) subtypes using International Classification of Diseases Ninth Revision, Clinical Modification (ICD9-CM) codes. A control group matched on sex, age, and index date of gout patients was randomly sampled with a ratio of 1:4 from the same database for comparison.

**Results:**

From 2002 to 2008, 28,769 gout patients who were older than 50 years old were identified, and 114,742 control patients was matched into the study. During follow-up, 7,119 patients developed dementia (1,214 with gout, and 5,905 without gout). After adjusting for age, sex, and relevant comorbidities, a Cox regression analysis showed that gout patients had a lower risk of developing non-vascular dementia (hazard ratio (HR): 0.77; 95% confidence interval (CI): 0.72 - 0.83; p < 0.001) and vascular dementia (HR: 0.76; 95% CI: 0.65 - 0.88; p < 0.001).

**Conclusions:**

Patients with gout have a lower risk of developing dementia. This phenomenon exists for both non-vascular and vascular types of dementia.

## Introduction

Dementia is one of the most commonly seen neurological diseases that have significant impact on public health. There are several types of dementia among which Alzheimer’s disease and vascular dementia are the two major types [1]. Vascular dementia has a strong association with stroke and metabolic syndrome. Recent studies also revealed association between Alzheimer’s disease and metabolic syndrome [2,3]. As gout and hyperuricemia are related to metabolic syndrome and cardiovascular diseases, this raises the question of whether patients with gout having higher risk of dementia [4]. However, it has long been proposed that uric acid has possible neuroprotective effects [5-7]. Some studies revealed that patients with gout have lower risks of developing Parkinson’s disease [8,9].A similar neuroprotective effect of uric acid has been found in animal models of multiple sclerosis [5]. It is still unclear whether uric acid has a protective effect in other neurologic degenerative diseases, such as dementia. Some more recent studies reveal that patients with Alzheimer’s disease tend to have lower central nervous system (CNS) uric acid levels [10], and higher serum urate level is associated with a reduced rate of cognitive decline in patients with mild cognitive impairment [11]. A cohort study by Euser *et al*. also demonstrated that higher uric acid levels are related to a decreased risk of dementia [12]. To determine the clinical implications of these findings, we examined the relationship between gout and dementia by analyzing a large population-based database.

## Methods

The study was approved by the Institutional Review Board of Taipei Veteran’s General Hospital (approval number: 2013-11-001CE). Informed consent of the study participants was not required because the dataset used in this study consists of de-identified secondary data released for research purposes.

### Study population

In Taiwan, the national health insurance (NHI) program launched in 1995 provides a universal and comprehensive health care system for all citizens. Registration to the NHI is obligatory for every citizen and hence its coverage rate is nearly 100%. The NHI program covers outpatient services, inpatient care, emergency medicine, Chinese medicine, dental care, and all other medically necessary services. Those who cannot afford the premium are subsidized so they can still be enroled. The NHI database contains the original claims data, including demographic information, and information on diagnosis, examinations, drug prescriptions, and operations.

The National Health Research Institutes acquired the original claims data for reimbursement from the Administration of National Health Insurance, Taiwan. After de-identification and some encryption processing, the data were released under the name of National Health Insurance Research Database (NHIRD) for research purposes. In addition, for ease of research and computing, NHIRD also provides a set of sampling files named the Longitudinal Health Insurance Database (LHID). In our study we used LHID 2005, which contains the original claims data of 1,000,000 beneficiaries randomly sampled from the entire population in 2005. During 2005 to 2006, there were about 25.68 million individuals in the whole NHI registry. There was no significant difference in the gender distribution, age distribution, or average insured payroll-related amount between the patients in the LHID 2005 and the original NHIRD (χ^2^ = 0.008, degrees of freedom (df) = 1, *P*-value = 0.931) [13]. The PostgreSQL Global Development Group (PostgreSQL) version 9.2 was adopted to manage the database. We also employed computer programs in structured query language (SQL) for initial data processing and sample selection.

### Ascertainment of gout

In the NHIRD, all diagnoses are documented using International Classification of Diseases Ninth Revision, Clinical Modification (ICD9-CM) codes. For the definition of gout cases, we used methods validated in the previous studies [14,15]. A patient was considered to have gout if he or she had ICD9-CM code 274 documented more than twice across outpatient visits. To ensure the accuracy of this definition, we carried out a sensitivity analysis. We tried to estimate the percentage of the people with gout who were receiving at least one prescription of medication for treatment of gout, including colchicine, allopurinol, benzbromarone, sulfinpyrazone, and probenecid. Seventy-five percent of the patients were prescribed at least one anti-gout drug; this ratio is very close to the numbers reported by other studies, suggesting that the inclusion of gout was valid [8]. As dementia is mainly a disease of the elderly, individuals younger than 50 years at the time of first gout diagnosis were excluded from the study. In our cohort, the incidence rates of gout per 1,000 person-years were 6.6 cases among male patients and 2.2 cases among female patients respectively. The values are nearly identical to that reported in other studies [8].

### Sampling of comparison group

**Figure 1:**
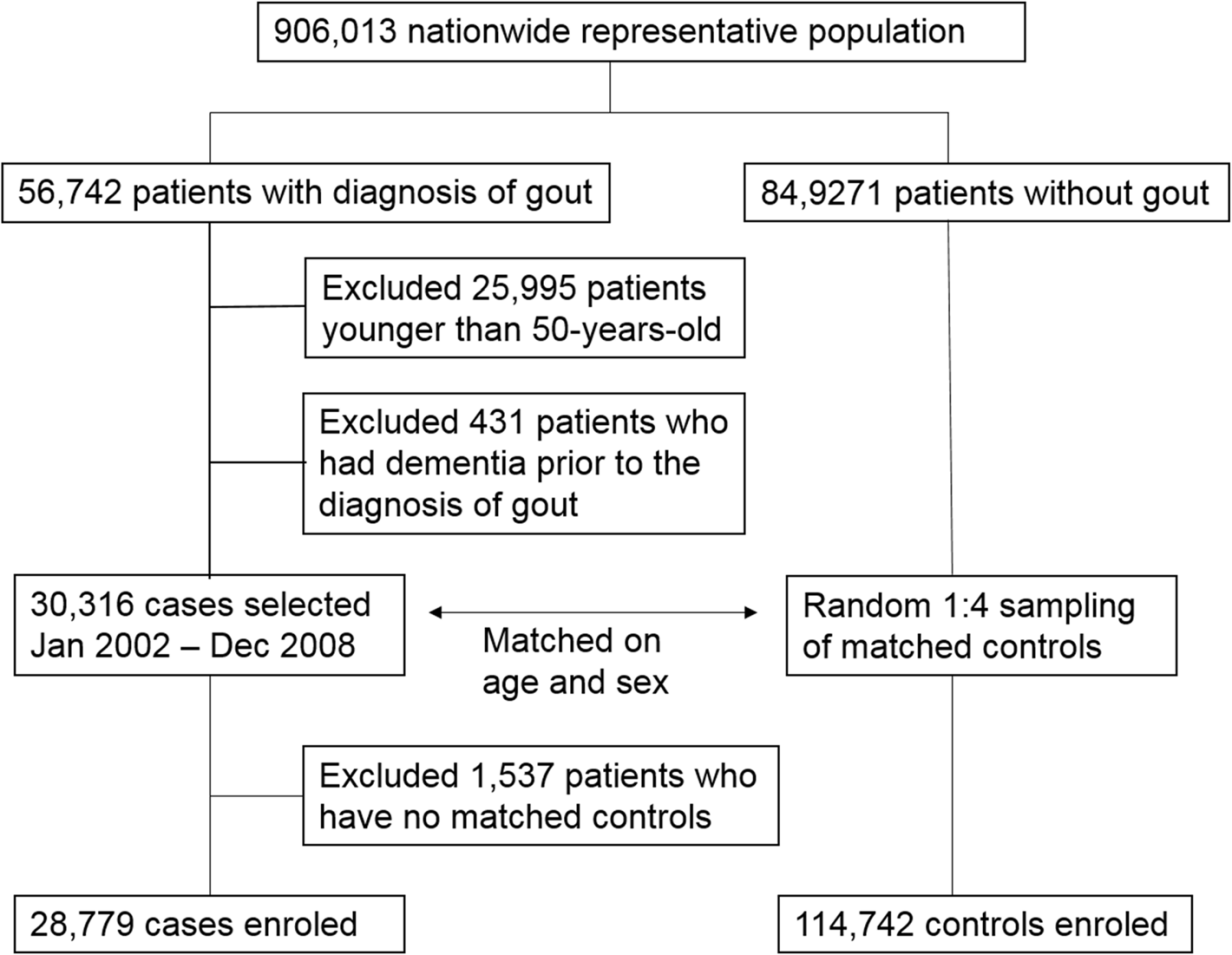
Flowchart of the included study sample. Among the included patients who had fewer than four controls, 80 patients had only one matched control, 45 patients had 2 controls, and 44 patients had 3 controls.

Up to four controls without gout, matched by age, sex, and index date for each gout case were randomly chosen from the same cohort. For each gout patient, we defined the date of the first recorded gout diagnosis as the index date. We then searched in the database for all age- and sex-matched controls who did not have gout on the patient’s index date. Next, we randomly sampled up to four controls for each individual from all eligible controls. The same procedure was performed repeatedly until all of them had found matched controls. During each iteration, controls that had already been selected previously were excluded. The baseline characteristics of the gout patients and their controls were based on the status on their index dates. Patients with gout for whom no matched controls could be found were excluded from the study. The recruitment process of the patients and their comparison group is summarized in Figure 1.

### Outcome measurement and ascertainment of dementia

Our primary outcome was the first recorded diagnosis of any type of dementia. The ascertainment of dementia was done by searching for ICD9-CM code 331.0 and 290.0-290.4 recorded in the claims. We further examined different subtypes of dementia. The patients with ICD9-CM code 290.4 series recorded in the claims were classified as having vascular dementia. All remaining dementia patients who did not belong to the vascular dementia group were defined as having non-vascular dementia. We also differentiated patients with Alzheimer’s disease from those with non-vascular dementia by searching for ICD9-CM code 331.0. With these definitions, the incidence density of dementia from the NHIRD during our study period was calculated as 2.02 per 1,000 person-years. All patients having dementia prior to the beginning of gout exposure or their index dates were excluded. The end of the study was 31 December 2008. Censoring was defined as death or the end of the study, whichever came first.

### Covariate assessment

For the ICD-9 CM codes used to ascertain other relevant comorbidities, we included diabetes mellitus (250), hyperlipidemia (272), heart failure (428), hypertension (401-405), coronary artery disease (410-414), chronic obstructive pulmonary disease (490-496), asthma (493.), ischemic stroke (433, 434, 436, 437.1), malignancy (140.0-208), chronic kidney disease (580-587), arrhythmia (427, 785.0, 785.1), and Parkinson’s disease (332). These comorbidities were tested in the univariate analysis and were adjusted for in the multivariate Cox regression analysis.

### Statistical analysis

The statistical analysis was conducted using SPSS Statistics version 20 (International Business Machines Corp., New York, NY, USA). In each group, patients’ data were described by frequencies and percentages for categorical variables, and by mean ± standard deviation for continuous variables. Categorical variables were compared using the chi-square test or Fisher’s exact test. Continuous variables were compared using the independent *t*-test. The Cox-proportional hazard model was used to estimate the relative risk of developing dementia in the adjusted analysis. Hazard ratios and 95% confidence intervals were computed. A two-tailed *P*-value <0.05 was considered statistically significant. To validate our results and avoid the potential bias from coding errors in dementia diagnosis, we conducted a sensitivity analysis by running the Cox regression test with a more strict definition of Alzheimer’s disease. The definition required not only ICD9-CM code 331.0 but also any documented prescriptions of medications used to treat Alzheimer’s disease, including donepezil, rivastigmine, galantamine, and memantine.

## Results

From 2002 to 2008, we ascertained 30,316 patients aged 50 years and over with first record of gout and no history of dementia. Of the gout patients 1,537 (5.1%) had no matched controls and were excluded. All of the excluded patients were male. The average age of the excluded patients was 68.8 ± 5.6 years, which is higher than the average age of the included patients (63.5 ± 9.7). Finally, 28,769 gout patients and 114,742 controls were enroled into the study. In the groups with and without gout, most of the patients were male (63.6% and 63.4%) and the average age was 63.5 ± 9.7 years in both groups. Compared with the non-gout group, individuals with gout had a higher frequency of comorbid medical conditions, including hypertension, hyperlipidemia, diabetes mellitus, coronary artery disease, heart failure, arrhythmia, chronic obstructive pulmonary disease, asthma, previous history of stroke, and chronic kidney disease. The mean follow-up duration was 4.3 ± 2.1 years for the gout cases and 4.4 ± 2.0 for the non-gout controls for all types of dementia. During the follow-up period, we identified 6,848 new cases of dementia (1,214 in the gout group and 5,634 in the non-gout group). The incidence of dementia in the gout and non-gout groups was 9.51 per 1000 person-years and 12.54 per 1000-person years, respectively. Detailed baseline characteristics and follow-up information on different dementia subtypes can be seen in Table 1.

**Table 1:** **Baseline characteristics and follow-up information of the gout and control groups**

	**No gout (n = 114,742)**	**Gout (n = 287,69)**	***P*** **-value**
Baseline characteristics			
Age	63.5 ± 9.7	63.5 ± 9.7	0.979
Male	72,642 (63.3%)	18,254 (63.4%)	0.724
Hypertension	38,947 (33.9%)	15,613 (54.3%)	<0.001
Hyperlipidemia	11,177 (9.7%)	6,566 (22.8%)	<0.001
Diabetes mellitus	18,968 (16.5%)	6,504 (22.6%)	<0.001
Coronary artery disease	16,937 (14.8%)	6,146 (21.4%)	<0.001
Heart failure	3,604 (3.1%)	1,533 (5.3%)	<0.001
Arrhythmia	11,678 (10.2%)	3,800 (13.2%)	<0.001
Chronic obstructive pulmonary disease	21,789 (19.0%)	6,590 (22.9%)	<0.001
Asthma	7,892 (6.9%)	2,698 (9.4%)	<0.001
Stroke	5,771 (5.0%)	1,611 (5.6%)	<0.001
Malignancy	5,484 (4.8%)	1,337 (4.6%)	0.341
Chronic kidney disease	5,128 (4.5%)	2,479 (8.6%)	<0.001
Parkinson's disease	1,296 (1.1%)	290 (1.0%)	0.077
Follow-up information			
All dementia types			
Follow-up duration	4.3 ± 2.1	4.4 ± 2.0	<0.001
New cases (incidence density)	5,905 (11.9)	1,214 (9.5)	
Vascular dementia			
Follow-up duration	4.4 ± 2.0	4.5 ± 2.0	<0.001
New cases (incidence density)	991 (2.0)	210 (1.6)	
Non-vascular dementia			
Follow-up duration	4.3 ± 2.0	4.5 ± 2.0	<0.001
New cases (incidence density)	4,914 (9.9)	1,004 (7.8)	
Alzheimer's disease			
Follow-up duration	4.4 ± 2.0	4.5 ± 2.0	<0.001
New cases (incidence density)	542 (1.1)	102 (0.8)	

Follow-up duration is expressed in person-years. Incidence density values are per 1000-person-years.

To assess the risks of developing dementia in both groups, both unadjusted univariate and adjusted multivariate Cox regression analysis were carried out. The results are summarized in Table 2. After adjusting for age, sex, and relevant comorbidities, patients with gout had a lower risk of developing dementia compared with the control group (hazard ratio (HR): 0.77, 95% CI: 0.72, 0.82, *P* <0.001). As dementia patients as a whole represent a heterogeneous group of various pathogeneses and clinical courses, we conducted a secondary analysis to estimate the risks of vascular and non-vascular dementia separately. The results are shown in Table 2. Among the dementia events, 1,154 were due to vascular dementia, and 5,694 due to non-vascular dementia. The patients with gout had lower risks of both vascular and non-vascular dementia. In the sensitivity analysis, gout patient still had lower risk of Alzheimer’s disease (HR: 0.76; 95% CI: 0.61, 0.95; *P* = 0.014), which remained consistent with our secondary analysis of non-vascular dementia.

**Table 2:** **Cox regression analysis results for gout and covariates on dementia**

	**Alzheimer's disease**		**Vascular dementia**		**Non-vascular dementia**		**All kinds of dementia**	
	**Hazard ratio (95% CI)**							
	**Unadjusted**	**Adjusted**	**Unadjusted**	**Adjusted**	**Unadjusted**	**Adjusted**	**Unadjusted**	**Adjusted**
Gout	0.74** (0.60, 0.91)	0.75* (0.61, 0.94)	0.83* (0.71, 0.96)	0.76** (0.65, 0.88)	0.79** (0.74, 0.85)	0.77** (0.72, 0.83)	0.79** (0.75, 0.85)	0.77** (0.72, 0.82)
Untreated gout	0.88 (0.62, 1.24)	0.89 (0.62,1.27)	0.92 (0.71, 1.19)	0.88 (0.86, 1.11)	0.97 (0.87, 1.09)	0.95 (0.85, 1.07)	0.96 (0.87, 1.07)	0.963 (0.84, 1.03)
Treated gout	0.67** (0.52, 0.88)	0.68** (0.52, 0.89)	0.79* (0.66, 0.95)	0.73** (0.60,0.88)	0.71** (0.65, 0.78)	0.69** (0.68, 0.75)	0.72** (0.67, 0.78)	0.69** (0.64, 0.75)
Other covariates								
Age	1.11** (1.10, 1.12)	1.11** (1.10, 1.12)	1.11** (1.10, 1.11)	1.10** (1.09, 1.10)	1.12** (1.11, 1.12)	1.11** (1.11, 1.12)	1.12** (1.11, 1.12)	1.11** (1.11, 1.11)
Male sex	0.75** (0.64, 0.88)	0.84* (0.71, 0.98)	1.11 (0.98, 1.25)	1.27** (1.13, 1.44)	0.86** (0.81, 0.90)	0.94* (0.89, 0.99)	0.89** (0.85, 0.94)	0.99 (0.94, 1.04)
Diabetes mellitus	1.29* (1.06, 1.58)	1.18 (0.96, 1.46)	1.87** (1.64, 2.13)	1.45** (1.26, 1.67)	1.50** (1.41, 1.59)	1.30** (1.23, 1.40)	1.56** (1.47, 1.65)	1.33** (1.25, 1.40)
Hypertension	1.44** (1.23, 1.69)	0.93 (0.78, 1.11)	2.30** (2.05, 2.57)	1.33** (1.16, 1.51)	1.67** (1.58, 1.75)	0.99 (0.93, 1.04)	1.76** (1.68, 1.84)	1.03 (0.98, 1.09)
Hyperlipidemia	0.93 (0.71, 1.20)	0.98 (0.74, 1.29)	1.15 (0.96, 1.37)	0.98 (0.81, 1.18)	0.91* (0.84, 1.00)	0.89* (0.81, 0.97)	0.94 (0.87, 1.02)	0.90* (0.83, 0.97)
Heart failure	1.56* (1.04, 2.33)	0.79 (0.52, 1.20)	2.50** (1.97, 3.18)	1.13 (0.88, 1.45)	2.17** (1.93, 2.42)	0.98 (0.87, 1.10)	2.22** (2.00, 2.45)	1.00 (0.90, 1.12)
Coronary artery disease	1.64** (1.35, 2.00)	1.10 (0.88, 1.37)	2.05** (1.80, 2.35)	1.11 (0.96, 1.30)	1.74** (1.63, 1.86)	0.99 (0.92, 1.07)	1.79** (1.68, 1.89)	1.04 (0.97, 1.10)
COPD	1.10 (0.90, 1.36)	0.67* (0.50, 0.87)	1..43** (1.24, 1.65)	0.82* (0.69, 0.98)	1.60** (1.50, 1.70)	1.02 (0.95, 1.10)	1.55** (1.47, 1.64)	0.97 (0.90, 1.04)
Asthma	1.28 (0.94, 1.73)	1.32 (0.90, 1.95)	1.32 (1.06, 1.65)	1.01 (0.78, 1.32)	1.44** (1.31, 1.59)	0.98 (0.88, 1.10)	1.41** (1.29, 1.54)	0.99 (0.89, 1.10)
Stroke	2.10** (1.6, 2.80)	1.16 (0.86, 1.57)	6.05** (5.24, 7.00)	2.98** (2.55, 3.49)	3.09** (2.85, 3.35)	1.55** (1.42, 1.69)	3.59** (3.234, 3.86)	1.80** (1.67, 1.95)
Malignancy	1.55* (1.09, 2.19)	1.18 (0.83, 1.67)	1.26 (0.96, 1.67)	0.85 (0.64, 1.12)	1.45** (1.29, 1.63)	1.00 (0.89, 1.13)	1.41** (1.27, 1.57)	0.96 (0.87, 1.07)
Chronic kidney disease	0.93 (0.62, 1.41)	0.73 (0.49, 1.11)	1.44** (1.13, 1.85)	0.95 (0.73, 1.22)	1.61** (1.45, 1.79)	1.17** (1.05, 1.30)	1.53** (1.43, 1.73)	1.12* (1.01, 1.23)
Arrhythmia	1.61** (1.27, 2.04)	1.16 (0.90, 1.50)	1.57** (1.31, 1.87)	0.91 (0.75, 1.10)	1.59** (1.47, 1.72)	1.01 (0.92, 1.10)	1.57** (1.47, 1.69)	0.98 (0.90, 1.06)
Parkinson's disease	4.81** (3.25, 7.12)	2.24** (1.50, 3.35)	6.45** (5.00, 8.32)	2.24** (1.72, 2.91)	6.76** (6.02, 7.59)	2.76** (2.45, 3.11)	6.94** (6.25, 7.71)	2.75** (2.47, 3.07)

These are the results of unadjusted univariate and multivariate Cox regression analysis. In adjusted analysis, all covariates listed in the table were entered into the model. No gout was the reference group for gout, treated gout and untreated gout groups. *P <0.05, **: *P* <0.01. COPD: chronic obstructive pulmonary disease.

## Discussion

The risks factors of Alzheimer’s disease and vascular dementia are both similar to those of cardiovascular diseases and cerebrovascular diseases, including hypertension and diabetes mellitus [16-18]. Given the fact that gout and hyperuricemia are both associated with cardiovascular diseases [4,19], it is more natural to postulate that gout might have positive correlation with dementia. However, the results from earlier studies with smaller samples and our population-based study all showed just the opposite direction [7,11]. This finding might support the possible neuroprotective effect of uric acid.

In our multivariate analysis, we also found that patients with older age, diabetes mellitus, stroke, chronic kidney disease, and Parkinson’s disease had significantly higher risks of dementia after adjusting for covariates. These results are consistent with existing literature as these factors are all known to be potential risk factors for dementia [16-18,20,21]. Some authors also propose that heart failure, hypertension, coronary artery disease, chronic obstructive pulmonary disease, asthma, and arrhythmia are associated with dementia, but there are also conflicting reports [18,22,23]. In our study, however, these factors were only associated with increased risks of dementia in univariate analysis. These differences might reflect the heterogeneity in study populations, study designs and outcome measurements. After adjustment for those confounding factors, we still found that the risk of dementia was about 25% lower in the group with gout.

Current understanding of the pathogenesis of Alzheimer’s disease is still limited, but evidence from existing studies has shown that the development of the disease is related to oxidative stress and deposition of β-amyloid [24]. Uric acid can act as an antioxidant and reduce oxidative stress [5,6,25]. It is a strong peroxynitrite (ONOO^−^) scavenger. A human study by Waring *et al*. also showed significant increase in serum free-radical scavenging capacity from baseline during uric acid infusion [26]. This might partly explain the potential neuroprotective effect. Besides, several hypotheses were proposed to explain the development of Alzheimer’s disease, including the amyloid cascade hypothesis and mitochondrial cascade hypothesis [27]. The amyloid cascade hypothesis assumes that Alzheimer’s disease is a primary amyloidosis originated from altered processing of amyloid precursor protein (APP) which drives production of amyloid-β (Aβ). The mitochondrial cascade hypothesis proposed that most Alzheimer’s disease is a secondary amyloidosis as a result of mitochondrial dysfunction. Brain mitochondrial dysfunction leads to imbalance of aerobic and anaerobic metabolism and triggered various cellular responses, including tau protein phosphorylation and cell cycle re-entry. Amyloidosis facilitates this alteration and finally neurodegeneration happens. In addition, apolipoprotein E (APOE) variations may affect cholesterol transport and affect amyloidosis [28]. APOE fragments also accumulate in mitochondria and affect its function [29]. Some studies showed that uric acid might preserve mitochondrial function [25]. A similar neuroprotective effect has been seen in studies of Parkinson’s disease and animal models of multiple sclerosis [5,30,31]. For example, uric acid is also related to repair of DNA damaged by free radicals in Parkinson’s disease [30]. Hence, it is possible that uric acid might provide a similar neuroprotective effect against the development of Alzheimer’s disease or other related dementia.

Vascular dementia being the second most common subtype of dementia accounting for 20% of all dementia was attributable to cerebrovascular pathologies [32]. In our study, the patients with gout also bear lower risk of developing vascular dementia, just like the observed trend in non-vascular dementia. Though the pathogenesis of vascular dementia is different from that of Alzheimer’s disease, they still share several common pathologic changes [33]. As in Alzheimer’s disease, amyloid angiopathy, oxidative stress, and inflammation were involved in the pathogenesis of vascular cognitive impairment (VCI). Deposits of Aβ in cerebral blood vessels are associated with VCI [32]. Because of this resemblance, it is also possible that uric acid might have neuroprotective potential in vascular dementia. Many existing studies have shown the relationship of oxidative stress and vascular dementia [32-34]. The study done by Back *et al*. demonstrated that markers of oxidative stress in the damaged white matter are associated with VCI [34]. Moreover, cerebral hypoperfusion is associated with white matter inflammation and oxidative stress in mouse models [32]. The animal study by Dong *et al*. revealed that administration of a superoxide scavenger in a mouse model prevented the development of white matter lesions and working memory deficit induced by chronic cerebral hypoperfusion [35]. This partly proves the concept that a superoxide scavenger might ameliorate the development of VCI caused by vascular factors. Whether uric acid can exert similar protection because of its scavenger property deserves further investigation.

In our study we noted that some patients had a history of stroke prior to the diagnosis of Alzheimer’s disease. It is possible that some of them actually had vascular dementia but were misclassified as having Alzheimer’s disease. Therefore we conducted another sensitivity analysis by reclassifying patients who had stroke prior to Alzheimer’s disease as having vascular dementia. Then we performed the same multivariate Cox regression test again. The results (data not shown) still revealed that patients with gout had lower risk of developing Alzheimer’s disease and vascular dementia.

It is also possible that the neuroprotective effect comes from uric acid-lowering medications rather than uric acid. To address this issue, we carried out another secondary analysis by separating patients with gout into treated and untreated subgroups. The definition of treated in our study was being prescribed with any kind of uric acid-lowering agents for more than 28 days. Patients with treated gout still had lower risks of dementia (HR: 0.68; CI: 0.63, 0.74; *P* <0.001). Those without treatment, however, did not show significant difference in risks of dementia compared to their controls. As our data were limited for insurance claiming and did not provide further information on treatment and test results, we could not attribute the results to the effect of the drugs. It was possible that patients in the untreated group had lower uric acid levels so they did not require drugs. Alternatively, it was also possible that they did not have gout but were misclassified. However, these plausible explanations would not be proven in our study. Nevertheless, from previous studies we know that drug noncompliance and nonpersistence are common among patients on uric acid-lowering treatment [36]. Patients with gout in general might still have higher serum uric acid levels. Moreover the neuroprotective effect of uric acid has already been shown in earlier studies [8]. Therefore the differences were more likely due to hyperuricemia itself rather than uric acid-lowering medications.

Although our results support the hypothesis that uric acid might have beneficial roles, such as a neuroprotective effect, our study has some limitations. In Taiwan, most of the citizens are not Caucasians, so whether the results of this study represent those in other ethnic groups requires further confirmation. In addition, all patient data are from the insurance claim data provided by NHIRD so potential misclassification might exist. Moreover, information on personal lifestyle including smoking status, body weight, body mass index, and laboratory data are not available. However, the merits of this study are the real-world data, large population size, and follow-up time.

## Conclusions

Patients with gout tend to have lower risk of developing dementia. Whether this effect comes from the proposed neuroprotective effects of uric acid is not proven in our study. However, if uric acid has neuroprotective potentials, clinicians might need to reevaluate the trade-off of reducing uric acid. Obviously, for optimal treatment of uric acid levels, more well-designed prospective trials are warranted.

## Abbreviations

APOE: apolipoprotein E
APP: amyloid precursor protein
Aβ: amyloid-β
HR: hazard ratio
ICD9-CM: International Classification of Diseases Ninth Revision, Clinical Modification
LHID: Longitudinal Health Insurance Database
NHI: national health insurance
NHIRD: National Health Insurance Research Database
VCI: vascular cognitive impairment
